# A Validation of the Equivalence of the Cell-Based Potency Assay Method with a Mouse LD_50_ Bioassay for the Potency Testing of OnabotulinumtoxinA

**DOI:** 10.3390/toxins16060279

**Published:** 2024-06-19

**Authors:** Yingchao Yang, Huajie Zhang, Liyong Yuan, Shuo Wang, Xiao Ma

**Affiliations:** National Institutes for Food and Drug Control, Beijing 102629, China; yangyc@nifdc.org.cn (Y.Y.);

**Keywords:** botulinum toxin type A, onabotulinumtoxinA, CBPA, equivalence, replacement

## Abstract

(1) Background: At present, the only potency assay approved in China for the in-country testing of botulinum toxin type A for injection products is the mouse bioassay (MBA). The Chinese market for neurotoxin products is rapidly expanding, but MBAs are subject to high variability due to individual variations in mice, as well as variations in injection sites, in addition to the limited number of batches tested for one MBA. Compared with the mLD_50_ method, the cell-based potency assay (CBPA) developed for the potency testing of onabotulinumtoxinA (BOTOX) by AbbVie not only does not use any experimental animals but also allows for significant time and cost savings. Due to the significant benefits conferred by the replacement of the mLD_50_ assay with CBPA in China, the CBPA method has been transferred, validated, and cross-validated to demonstrate the equivalence of the two potency methods. (2) Methods: The differentiated SiMa cells were treated with both BOTOX samples and the reference standard, and the cleaved SNAP25_197_ in the cell lysates was quantified using Chemi-ECL ELISA. A 4-PL model was used for the data fit and sample relative potency calculation. The method accuracy, linearity, repeatability, and intermediate precision were determined within the range of 50% to 200% of the labeled claim. A statistical equivalence of the two potency methods (CBPA and mLD_50_) was initially demonstrated by comparing the AbbVie CBPA data with NIFDC mLD_50_ data on a total of 167 commercial BOTOX lots (85 50U lots and 82 100U lots). In addition, six lots of onabotulinumtoxinA (three 50U and three 100U) were re-tested as cross-validation by these two methods for equivalence. (3) Results: The overall assay’s accuracy and intermediate precision were determined as 104% and 9.2%, and the slope, R-square, and Y-intercept for linearity were determined as 1.071, 0.998, and 0.036, respectively. The repeatability was determined as 6.9%. The range with the acceptable criteria of accuracy, linearity, and precision was demonstrated as 50% to 200% of the labeled claim. The 95% equivalence statistic test using margins [80%, 125%] indicates that CBPA and mLD_50_ methods are equivalent for both BOTOX strengths (i.e., 50U and 100U). The relative potency data from cross-validation were within the range of ≥80% to ≤120%. (4) Conclusions: The CBPA meets all acceptance criteria and is equivalent to mLD_50_. The replacement of mLD_50_ with CBPA is well justified in terms of ensuring safety and efficacy, as well as for animal benefits.

## 1. Introduction

Botulinum neurotoxins (BoNTs) are the most potent natural toxins known [1,2] to humankind. Seven distinct BoNT serotypes (A–G) have been reported, and they block acetylcholine release from presynaptic terminals at neuromuscular junctions, thereby causing flaccid paralysis [2,3,4]. The 50 kDa light chain (LC) of BoNTs is made of zinc metalloproteases and is connected by disulfide bonds to the 100 kDa heavy chain [5]. LCs cleave one of their target proteins at presynaptic termini and thereby inhibit synaptic transmission. These cleaved proteins include the synaptosomal-associated protein of 25 kDa (SNAP25), the vesicle-associated membrane protein, and syntaxin. Serotype BoNT/A and BoNT/E both target SNAP25. However, muscle paralysis caused by BoNT/A can last for several months, whereas the effects of BoNT/E are relatively short-lived [6].

The use of botulinum toxin A (BoNT-A) in medicine has increased markedly since the first applications during the mid-1980s [7]. Current aesthetic uses of BoNT-A include the treatment of glabellar lines, forehead wrinkles, periorbital and perioral lines, platysmal bands, horizontal neck lines, and the masseter, among its many other applications [8]. The net revenue of onabotulinumtoxinA (BOTOX^®^ and BOTOX^®^ COSMETIC) reached USD 748 million and USD 620 million in the third quarter of 2023 for therapeutic and aesthetic indications, respectively [9]. Indeed, according to the American Society of Plastic Surgeons, 13.2 million minimally invasive aesthetic procedures were carried out in the United States during 2020, and BoNT-A procedures were used in 4.4 million out of a total of 13.2 million and hence represent the most commonly used of all BoNT/A products [10]. Of almost 14 million aesthetic treatments performed by members of the American Society for Dermatologic Surgery in 2019, 2.3 million of these were BoNT-A procedures [11].

Five BoNT-A products have been approved in China for therapeutic indications: onabotulinumtoxinA (BOTOX^®^, AbbVie Inc., USA), lanbotulinumtoxinA (Hengli^®^, Lanzhou Biotechnique Development Co. Ltd., China), abobotulinumtoxinA (Dysport^®^, Ipsen Biopharm Limited, France), letibotulinumtoxinA (Letybo^®^, Hugel Inc., Korea), and IncobotulinumtoxinA (Xeomin^®^, Merz Pharma GmbH & Co. kGaA, Germany). The use of BoNT/A in therapeutic and aesthetic indications has also rapidly increased in China. From 2017 to 2021, the market size of botulinum toxin products in China increased from CNY 1.9 billion to CNY 4.6 billion, with a compound annual growth rate of 25.6% [12].

Currently, the only potency assay approved in China for the in-country testing of botulinum toxin type A products for injection is the mouse LD_50_ bioassay. However, the mLD_50_ method is known to be susceptible to individual differences in animals, experimental conditions, and injection sites and angles in mice, and the number of mice tested is directly due to the high variability of the MBA. A lot of work has been carried out over decades by many groups worldwide to find a suitable replacement. Compared with the mLD_50_ method, the clostridium botulinum neurotoxin serotype A cell-based potency assay (CBPA) does not use experimental animals and also saves significant time and cost [13,14]. As was well summarized in a review paper [15], a number of groups in both academia and industry believe that cell-based assays have a strong potential to replace the MBA in terms of BoNT potency determination in pharmaceutical formulations; they can also help to identify suitable inhibitors while reducing the number of animals used.

AbbVie CBPA uses a BB10 clonal isolate of the SiMa human neuroblastoma cell line obtained from the German cell bank repository, Deutsche Sammlung von Mikroorganismen und Zellkulturen. With this cell line, the assay mimics the in vivo mechanism of the actions of BoNT/A, including binding to the cell-surface receptors, internalization, translocation of the light chain (LC) into the cytosol, and the proteolytic cleavage of the synaptosome-associated protein of 25 kilodaltons (SNAP25_206_) by the BoNT/A LC endopeptidase between amino acids 197 and 198, resulting in 197-amino acid SNAP25 protein or SNAP25_197_. The monoclonal antibody 2E2A6, which was developed by AbbVie to specifically recognize SNAP25_197_, is used to quantify SNAP25_197_. The good sensitivity of the BB10 cells to BoNT/A, the specificity of the 2E2A6 antibody for SNAP25_197_, and the electrochemiluminescence enzyme-linked immunosorbent assay (ECL ELISA) provide the sensitivity necessary to quantify the biologically relevant potency of the BoNT/A drug product. It is currently being used as an alternative potency testing method to mLD_50_ in the release and stability testing of the BOTOX drug produced by AbbVie. 

Due to the desire for the replacement of the mLD_50_ assay with CBPA for in-country testing in China, the BOTOX CBPA method transfer, validation, and based cross-validation against mLD_50_ were executed by Chinese National Institute of Food and Drug Control. 

## 2. Results

### 2.1. Accuracy 

A qualified Working Potency Reference Standard lot, WPRS04 (nominal potency of 103 U/vial), was used in validation studies of CBPA performance. It was manufactured in the same way as commercial 100U BOTOX^®^ lots and thus reconstituted for CBPAs in the medium as commercial BOTOX^®^ lots. WPRS04 was used as both the reference standard and for test samples prepared at five target potency levels (50%, 70%, 100%, 130%, and 200%) by two analysts, to a minimum of n = 3 of CBPA results for each potency level, over different testing weeks. The acceptance criterion was within 85% to 115% recovery of the target potency level.

Accuracy was calculated using Equation (1), where the relative potency value obtained from CBPA results is divided by the target relative potency value, expressed as the ratio of the preparation to the nominal level (50%, 70%, 100%, 130%, and 200%).

The X-axis is the log10-based transformation of nominal BOTOX^®^ concentration in U/mL.

**Figure 1:**
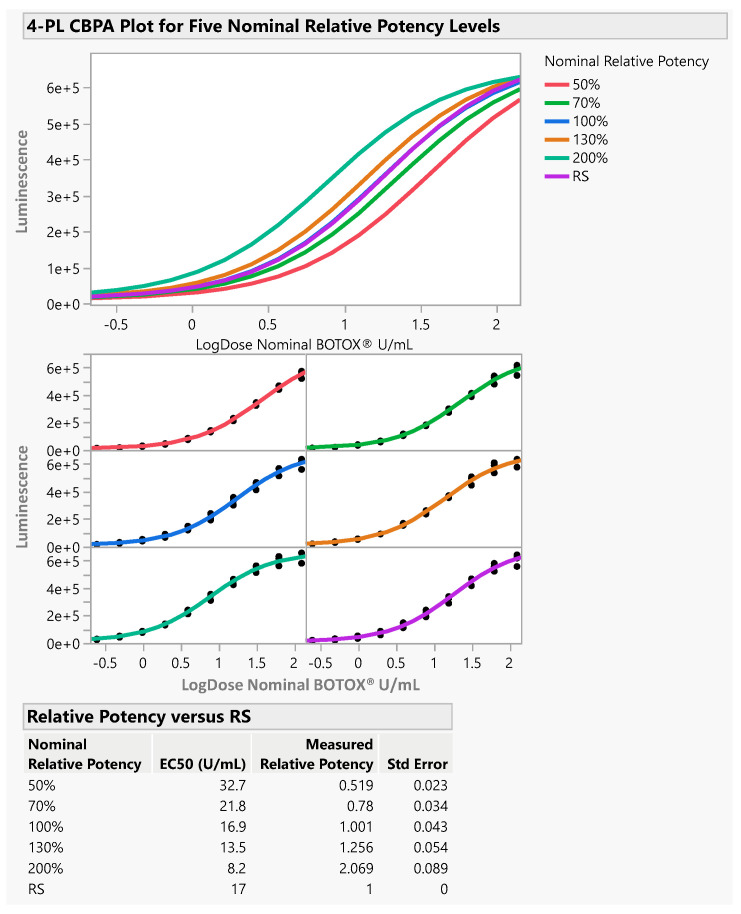
Representative 4-PL CBPA plot for five nominal relative potency levels.

As shown in Table 1 and Figure 1, below, the accuracy results at each level fell within the range of 80% to 115% of the nominal values; the acceptance criteria were met. The overall method accuracy was 104%, which also meets the acceptance criteria. Figure 1 shows one round of accuracy results by one analyst.

### 2.2. Intermediate Precision 

Intermediate precision was evaluated by calculating the relative standard deviation (Equation (2)) between targeted potency levels, analysts, and assay dates. The acceptance criterion for %RSD (relative standard deviation) was ≤15%, and the overall %RSD ≤ 10%.

As shown in Table 2, below, the assay’s intermediate precision met the acceptance criterion. The overall intermediate precision %RSD was 9.2%, which met the acceptance criterion of ≤10%.

### 2.3. Linearity

Linearity was determined using accuracy and intermediate precision test data. The acceptance criterion for linearity is that the slope of the plot of expected vs. measured values must be ≥0.80 ≤ 1.20, R^2^ ≥ 0.95. The result of RMSE (Root Mean Square Error) and the Y-intercept should be reported for information only.

**Figure 2:**
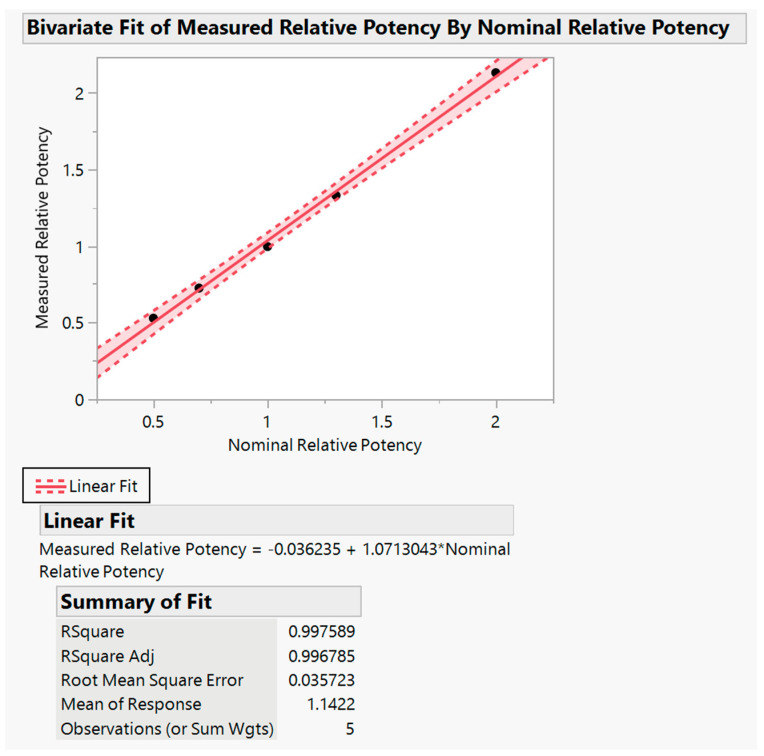
Summary of method’s linearity results. (The black dots are five nominal relative potency, i.e., 50%, 70%, 100%, 130% and 200%.).

As shown in Figure 2, below, the slope of the plot of measured potency against expected potency was determined as 1.071, and the R^2^ value was determined as 0.998, both of which met the pre-set acceptance criteria. The Y-intercept and RMSE were determined as 0.036 and 0.036, respectively.

### 2.4. Repeatability 

Repeatability was assessed by testing the CBPA results to a minimum of n = 6, and pooled WPSR04 was tested as both the reference standard and the sample by one analyst in a single test session. The acceptance criterion for repeatability was RSD ≤ 10%.

As shown in Table 3, below, the repeatability (RSD) was determined as 6.9%, which met the acceptance criterion of ≤10%. The details of the repeatability results can be seen in Table 3.

### 2.5. Equivalence Test 

**Figure 3:**
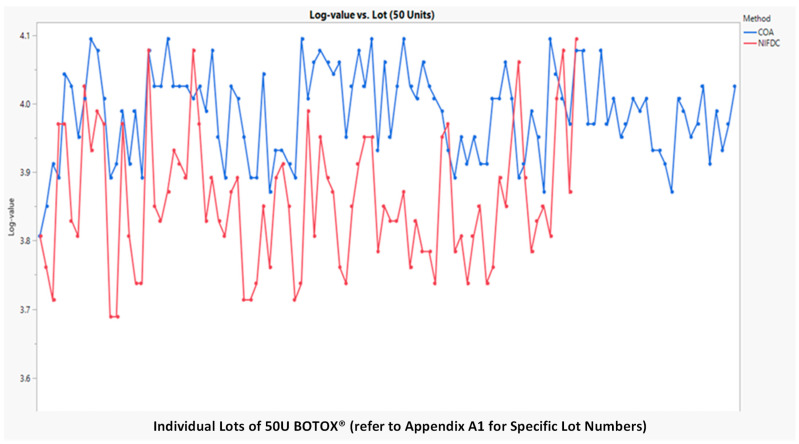
Log-potency values (Y-axis) vs. lot number (X-axis) for the two methods at 50 units.

**Figure 4:**
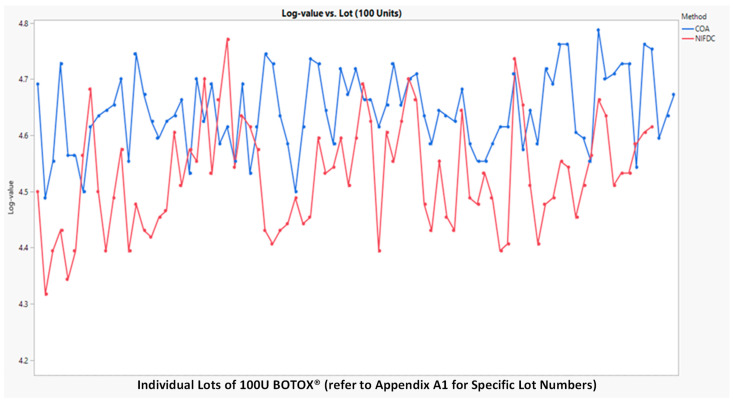
Log-potency values (Y-axis) vs. lot number (X-axis) for the two methods at 100 units.

A statistical equivalence test of the two potency assays was first performed on BOTOX^®^ lots at two nominal potency strengths of 50U (Figure 3) and 100U (Figure 4). The methods were compared using orthogonal regression to assess whether the slope between them was significantly different from one and mixed models (with lot as random) to calculate any offset between the methods under various levels of variation for the two methods. The FDA guidance provided in “Statistical Approaches to Establishing Bioequivalence Guidance for Industry (2022)” was followed by a statistical evaluation of equivalence between CBPA and mLD_50_ methods.

The statistical equivalence of the two assays was supported by the existing total of 167 commercial batch test data. The dataset used for method comparison includes Certificate of Analysis (COA) CBPAs and mLD_50_ potency values conducted by the Chinese NIFDC, tested side by side. 

The 95% equivalence test using margins [80%, 125%] indicates that CBPAs (performed by AbbVie) and mLD_50_ (performed by Chinese NIFDC) methods are equivalent for both potency levels.

Table 4 and Table 5, below, show the results of the equivalent test from the mixed effects model. The log-transformed potency value vs. lot number for the COA CBPA and NIFDC mLD50 methods are plotted in Figure 3 for 50 units and Figure 4 for 100 units, respectively. In general, NIFDC mLD_50_ had lower potency values compared with the COA CBPA method. The geometric mean difference was rather small, as shown in Table 5. The orthogonal fit was used to compare the two methods in 50 and 100 units, respectively. The 95% two-sided confidence interval for slope included 1.0 (see Table 4), suggesting that no proportional error was detected. Hence, the mixed effects model can be used for the method comparison. Based on the analysis result, the ratio of geometric means of CBPA vs. mLD50 was 113.84 for 50 units and 112.43 for 100 units. The 90% confidence interval for the ratio of geometric potency means for COA CBPA vs. mLD_50_ was (111.9, 115.83) for 50 units and (110.32, 114.58) for 100 units, respectively. Both intervals fell within the (80%, 125%) range. The two potency assays were statistically equivalent for both nominal potency levels within the range assessed. The method variance for CBPAs (0.004 to 0.005) was less than the variation for mLD_50_ (0.009 to 0.010). CBPAs had a 38% lower standard deviation compared to mLD_50_. The related SAS code and report can be found in Appendix A.

Cross-validation was performed on six commercial BOTOX lots (three at 100U and three at 50U) to confirm the equivalence between CBPA and mLD_50_ methods, both of which were performed by Chinese NIFDC (Table 6). The equivalence acceptance criteria were the “results of both potency methods passing the release specifications of 80% to 120% of labeled claim”. The CBPA results were produced by AbbVie and Chinese NIFDC independently.

As shown in Table 6, above, each of the results met the pre-set acceptance criteria of ≥80% and ≤120% of the labeled claim. Therefore, it was concluded that CBPA and mLD_50_ are equivalent in determining BOTOX potency at both 50 U/vial and 100 U/vial nominal potency. More data can be found in Appendix A.

## 3. Discussion

The first CBPA method to determine the potency of the BoNT/A product was developed by Allergan (now AbbVie) [16]. It obtained regulatory approval from the United States Food and Drug Administration in June 2011 and subsequently from the European Union in February 2012. A cell-based assay has also been developed for incobotulinumtoxinA (Xeomin^®^) [17] and for abobotulinumtoxinA (Dysport^®^, Azzalure^®^) [18]. 

The mLD_50_ assay, which has been the gold standard for BoNT/A potency testing and the only method approved by China for Botulinum Toxin Type A for injection products, has many intrinsic disadvantages, including a susceptibility to high variability in animals used in mLD_50_ testing, expensive facilities, variable injection sites and angles in mice, and a limited number of sample batches that can be tested at one time [19]. Considering that the Chinese market for neurotoxin products is rapidly expanding, the mLD_50_ method is becoming less able to meet the growing demand for product testing, likely resulting in delays in the release of test reports, and other related problems. Furthermore, the use of animals in lethality testing has become more and more unacceptable to the public due to ethical concerns. Compared with the mLD_50_ method, however, the CBPA method not only does not use any experimental animals but also saves significant time and cost. It has the potential to completely replace mLD_50_ assays for the batch release of BoNT/A products. 

Due to the understandable desire for replacing mLD_50_ assays with CBPA in China, the AbbVie BOTOX CBPA method’s transfer, validation, and cross-validation against mLD_50_ to demonstrate the equivalence of these two potency methods were designed and executed by Chinese NIFDC. The experimental strategies for CBPA validation and equivalence were based on the relevant requirements as described in the ICH Q2 R1 guidelines [20] and the Chinese Pharmacopoeia, 2020 Edition, with thorough consideration of the experience gained from method development. The overall strategy consists of three parts: (1) CBPA method validation to determine assay parameters such as range, accuracy, linearity, repeatability, and intermediate precision. When all parameters meet the acceptance criteria, the method is considered validated. (2) Demonstration of the equivalence between CBPA and mLD_50_ methods by comparing the data obtained by the NIFDC mLD_50_ method as in-country testing and with the CBPA data obtained by Allergan as GMP release testing on the same set of BOTOX^®^ samples, i.e., 85 batches for 50 U and 82 batches for 100 U. (3) Confirmatory equivalence cross-validation between the CBPA method validated by Chinese NIFDC and the Chinese NIFDC mLD_50_ method conducted by NIFDC on a total of six commercial BOTOX lots (3 at 100 U and 3 at 50 U) which were previously released by Allergan. If all the results fulfill the corresponding acceptance criteria for each of these three strategy parts, the CBPA is considered acceptable as a replacement for mLD_50_.

Since the CBPA method includes many steps and takes approximately two weeks (seven-day mitotic propagation after the thawing of frozen vials in culture flasks, three-day differentiation in 96-well plates, and three-day CBPA) to produce the final test results, the assay performance can potentially be affected by several factors, such as cell passage numbers, antibody shelf life, and chemiluminescence substances. The capture and detection antibodies used in AbbVie CBPA are stored at −20 °C. To ensure the consistent performance of these antibodies, annual qualification and re-qualification need to be performed. The antibodies are considered qualified or re-qualified if the signal-to-noise ratio meets the acceptance criteria of ≥15. Further, different pharmaceutical companies perform toxin CBPA in various formats, including using different cells, detection methods, and statistics for data analysis. The equivalence of any two CBPA methods of different companies will not be assessed and the replacement of mLD_50_ with company A’s CBPA for company B’s product will not be realized.

In this study, the AbbVie CBPA for onabotulinumtoxinA was proven to be equivalent to the mLD_50_ assay for determining the potency of BOTOX lots as in-country testing by statistical comparability assessments of cross-validation results. We believe that this CBPA method can be used as a replacement for mLD_50_ in the in-country testing of commercial BOTOX lots.

While working on the validation and cross-validation of AbbVie CBPA, we noticed that Ipsen published a paper in Toxins describing a CBPA method for determining the potency of abobotulinumtoxinA (Dysport^®^ and Azzalure^®^ for powder formulation and Alluziennce^®^ for liquid formulation) [18]. It is really encouraging to see that more and more academic as well as commercial entities are replacing animal-based methods with cell-based methods for the potency testing of botulinum neurotoxin in the 3R spirit. Since all the methods are different from each other, the following sections are intended to compare the two CBPA methods, one from Ipsen and the other from AbbVie, which could serve as a potential navigator for CBPA developers. As a starter, the Ipsen CBPA relies upon a genetically engineered mouse neuroblast cell line, Neuro-2a, that expresses a reporter protein containing the full-length SNAP-25 flanked by cyan fluorescent protein (CFP) and yellow fluorescent protein (YFP). When these engineered cells are incubated with BoNT/A, the BoNT/A light chain metalloprotease enters the cytosol and cleaves the reporter, resulting in the release of a C-terminal reporter fragment into the cytosol that contains residues 198–206 of SNAP-25 and YFP. That fragment is degraded by the cell, resulting in a BoNT/A dose-dependent loss of yellow fluorescence [21]. Therefore, the Ipsen CBPA is easy to perform; for example, by following exposure to the BoNT/A serial dilutions, the raw data can be obtained by simply reading the CBPA assay plates in a fluorescence plate reader. On the other hand, the AbbVie CBPA requires a chemi-ECL ELISA to quantify the endogenous 197-amino acid SNAP25 protein cleaved by the BoNT/A light chain between amino acids 197 and 198. Not only would the chemi-ECL require additional hands-on steps compared with the Ipsen method, but the chemi-ECL ELISA also requires the maintenance of critical agents such as capture and detection antibodies.

However, in Ipsen’s CBPA, the endogenous SNAP25 protein is also expressed and thus could compete with the CFP-SNAP25-YFP fusion protein for the BoNT/A light chain metalloprotease. In addition, the Ipsen CBPA assay window depends not only on how much the fusion protein is cleaved but also on how fast the cleaved YFP is degraded by the cells. In AbbVie CBPA, however, as soon as the endogenous SNAP25 protein is cleaved by the BoNT/A light chain, the 197-amino acid becomes a detectable molecule for chemi-ECL ELISA. Furthermore, the SNPA25 protein flanked by CFP and YFP may not be as accessible to the BoNT/A light chain as the endogenous SNPA25 due to the potential physical hindrance of CFP and YFP. The combination of the above properties of Ipsen CBPA could explain the large difference in the assay window between Ipsen and AbbVie CBPA. The typical assay window for AbbVie CBPA is over 100,000-fold (signal of highest concentration: signal of lowest concentration), whereas the Ipsen CBPA seems to have an assay window of less than 2-fold [22]. It is generally acknowledged that narrow assay windows could be a limitation to assay performances in areas such as sensitivity and precision. It may also be worth pointing out that the Neuro-2a cell line used by Ipsen is mouse-originated and the SiMa BB10 cells used by AbbVie are human-originated.

As BoNT/E has emerged as a promising drug candidate due to the quicker onset of action and shorter duration of effect when compared to BoNT/A and BoNT/B [23,24,25,26], CBPA can also be applied for the detection and development of BoNT/E and other BoNT serotypes.

## 4. Conclusions

The AbbVie BOTOX CBPA has been demonstrated to meet all validation and cross-validation acceptance criteria, and equivalence was confirmed between the CBPA and mLD_50_ by statistical assessment. The replacement of the mLD_50_ assay by CBPA is reasonable and feasible.

## 5. Materials and Methods

### 5.1. Sample Preparations 

The reconstitution media and the reconstitutions of each of the five potency levels were prepared.

After reconstitution, nine 2-fold serial dilutions were performed on the samples using a dilution medium to generate a range of ten concentrations for treating BB10 cells. Five potency levels were prepared using WPRS04, according to Table A2.

For accuracy testing, the 3-plate layout provided in the method was used to determine the relative potency of five individually prepared test samples compared to the reference standard.

For the repeatability study, a single analyst performed multiple measurements on a sample prepared at the nominal 100% level during a single experiment. The 3-plate layout in the method can be used to generate up to five test results. To obtain a minimum of six results for the repeatability study, the use of two sets of the 3-plate layout would generate a total of ten results.

### 5.2. The CBPA Assay Methodology *[16]*

#### 5.2.1. Cell Growth and Differentiation (Days 1–11)

After thawing and initial cell culture in tissue culture flasks, the cells were transferred to type I collagen-coated plates and culture for approximately 72 h under conditions that included trisialoganglioside (GT1b) and neuronal supplement to enhance cell sensitivity to neurotoxin uptake.

#### 5.2.2. Cell Treatment and Accumulation of SNAP25_197_ (Days 11–15)

Cells were treated with RS and test articles, both of which were in 9060X formulation, for 24 h. During this period, neurotoxin was bound to cell surface receptors and was internalized, and the peptide cleavage domain of the light chain (LC) was translocated into the cytosol, where it cleaved SNAP25 between amino acids 197 and 198. The cells were cultured for an additional 72 h in a fresh medium to accumulate cleaved SNAP25_197_.

#### 5.2.3. Sandwich ELISA for the Quantification of SNAP25_197_ (Days 15 and Day 16)

The cells were lysed and the lysates containing the cleaved SNAP25_197_ were quantified using the sandwich enzyme-linked immunosorbent assay (ELISA) technique. This ELISA method utilizes a polyclonal capture antibody that recognizes both cleaved and un-cleaved SNAP25 in conjunction with the detection antibody 2E2A6 (conjugated with horseradish peroxidase (HRP)) specific to SNAP25_197_. The capture antibody binds to SNAP25 contained within the cell lysates onto the ELISA plate and the detection antibody binds to the cleaved SNAP25_197_ on the plate. After stimulation with hydrogen peroxide substrate, the detection antibody conjugated with HRP generated a transient luminescent signal. Luminescence was measured in luminescence units (LUM) using the Synergy Neo plate reader equipped with a luminescence detector.

#### 5.2.4. Data Analysis

Statistical analysis software JMP SAS was employed for data analysis. The raw data of both standard and test samples were fitted to a four-parameter logistic (4-PL) parallel model. Subsequently, the potency of the test sample was calculated relative to the standard. The weighted factor to the Botox CBPA assay was 1.64.

The BB10 cells used during the method validation were from Passage 19 (P19) and Passage 20 (P20) cell lines.

### 5.3. CBPA Assay Method Validation 

Four tests were designed and performed each week. In each test of the first three weeks, two analysts each tested each of the five potency levels of Botox^®^ once using the CBPA method, for a total of six measurements over three weeks, to validate the accuracy, linearity, range, and intermediate precision of the assay. For each test, each analyst measured the response on three 96-well plates to obtain one reportable potency result for each potency level. Six reportable potency results should be obtained for each potency level in total over 3 weeks. A minimum of 3 results meeting the method validity criteria per potency level are required for data analysis. In the repeatability test in the fourth week, one analyst performed the test. Six 96-well plates were used in the repeatability test, and a total of 10 sets of data were obtained. A minimum of 6 valid data points for each test level in the repeatability test are required for data analysis. Through reconstitution procedures, reference standards were reconstituted to 50%, 70%, 100%, 130%, and 200% of the labeled potency, serving as test samples. A summary of the study execution is provided in Table A3.

(1) $$\mathrm{Accuracy}\;\left(\%\right)=\frac{\mathrm{CBPA}\;\mathrm{Relative}\;\mathrm{Potency}\;\mathrm{Value}\;}{\mathrm{Target}\;\mathrm{Relative}\;\mathrm{Potency}\;\mathrm{Value}\;}\times 100\%$$

RSD (%) = (Standard Deviation/Mean) × 100 (2)

### 5.4. Cross-Validation Methodology 

The cross-validation involved a comparison between CBPA and mLD_50_ methods based on six batches (three batches for 50U and three batches for 100U) of products previously released by Allergan to confirm the equivalence between the CBPA method and the mLD_50_ method according to Table 6 and Table A1 in Appendix B. If all three potency assay results mentioned above met the release criteria defined in the approved specifications for Botox^®^, then the CBPA method and mLD_50_ method conducted by NIFDC can be considered equivalent.

## Figures and Tables

**Table 1:** Summary of accuracy results.

Nominal Relative Potency (%)	Measured Relative Potency (%)	Accuracy (%)	Mean Accuracy (%)	% CV (Precision per Level)	Overall Accuracy	Overall Intermediate Precision (%CV)
50 (n = 5) ^a^	54	108	105.2	5.3	103	9.2
	52	104				
	48	96				
	54	108				
	55	110				
70 (n = 5) ^a^	64	91	104	11.8		
	78	111				
	63	90				
	82	117				
	76	109				
100 (n = 5) ^a^	90	90	100	7.3		
	100	100				
	95	95				
	105	105				
	108	108				
130 (n = 5) ^a^	123	95	102	12.2		
	126	97				
	118	91				
	158	122				
	140	108				
200 (n = 5) ^a^	241	121	107	10.2		
	207	104				
	181	91				
	220	110				
	216	108				

^a^ n represents the number of CBPA results.

**Table 2:** Summary of intermediate precision results.

Parameter	Test Level					
	50%	70%	100%	130%	200%	Overall
Relative standard deviation (%)	5.3	11.9	7.0	12.3	10.2	9.2

**Table 3:** Summary of method’s repeatability results.

Nominal Relative Potency (%)	Measured Relative Potency (%)	Accuracy (%)	Mean Accuracy (%)	% CV (Precision per Level)	Relative Standard Deviation (%)
100 (n = 10) ^a^	98	98	102	1.6	6.90
	97	97			
	99	99			
	95	95			
	96	96			
	106	106			
	116	116			
	111	111			
	102	102			
	99	99			

^a^ n represents the number of CBPA results.

**Table 4:** Orthogonal fit table.

Units/Vial	Slope	LowerCL	UpperCL	Alpha
50	1.411	0.794	2.507	0.05
100	1.368	0.436	4.289	0.05

**Table 5:** Summary statistics and equivalence test results between CBPA and mLD50 potency assays.

Units/vial	Test Group	Geometric Means		Ratio of Geometric Means		
	DF	CBPA ^1^	mLD50 ^2^	CBPA/mLD50	90% LowerCL	90%UpperCL
50	109	3.985	3.856	113.84	111.90	115.83
100	84	4.644	4.527	112.43	110.32	114.58

^1^: conducted by AbbVie; ^2^: conducted by NIFDC.

**Table 6:** Summary cross-validation results of six batches.

Test Batch	Nominal Potency ^a^	CBPA ^1^	CBPA ^2^	mLD_50_^1^
C7278C3	100	106	95	93
C8679C3	100	97	107	112
C8486C3	100	104	103	111
C8441C2	50	51	57	55
C8399C2	50	50	49	58
C8596C2	50	46	55	57

Here, ^a^ shows the result data in this table, expressed in “U/vial”; ^1^ denotes the test performed by NIFDC; ^2^ denotes the test performed by Allergan.

## Data Availability

The data presented in this study are available in this article and Supplement Materials.

## Supplementary Materials

The following supporting information can be downloaded at: https://www.mdpi.com/article/10.3390/toxins16060279/s1.

## Appendix A

SAS Code SAS, Outputs and JMP 16.1 Outputs

SAS code

ods graphics on;

proc mixed data = dat;

by Unit;

class Lot Method;

model Log value = Method/cl alpha= 0.1;

lsmeans Method/cl alpha = 0.1 pdiff = control(“NIF”);

Repeated Method/type = un subject = Lot;

ods output LSMeans = lsm Diffs = lsmdiff;

ods exclude IterHistory NObs ModelInfo;

run;

data Estimates; set lsmdiff;

if Unit = 50 then do;

geomean_ratio = exp (Estimate)*100;

LowerCL = exp((Lower)) *100;

UpperCL = exp((Upper)) *100;

end;

if Unit = 100 then do;

geomean_ratio = exp (Estimate)*100;

LowerCL = exp((Lower)) *100;

UpperCL = exp((Upper)) *100;

end;

run;

data Estimates_lsm; set lsm;

geomean = Estimate;

LowerCL = (Lower);

UpperCL = (Upper);

run;

title “90% CL for Ratio of Geometric Means”;

proc print data = Estimates noobs;

var Unit DF geomean_ratio LowerCL UpperCL;

run;

title “Geometric Mean and 95% CI for Each Method”;

proc print data = Estimates_lsm noobs;

var Unit Method DF geomean LowerCL UpperCL;

run;

title;

---50 Unit

---100 Units

SAS Output

**The Mixed Procedure**

**Unit = 50**

**Class Level Information**		
**Class**	**Levels**	**Values**
Lot	110	C4409C2 C4722C2 C5016C2 C5483C2 C5554C2 C5847C2 C6178C2 C6311C2 C6676C2 C6677C2 C6695C2 C6717C2 C6718C2 C6724C2 C6749C2 C6768C2 C6769C2 C6782C2 C6793C2 C6803C2 C6816C2 C6831C2 C6844C2 C6849C2 C6851C2 C6928C2 C6941C2 C6944C2 C6956C2 C6996C2 C7080C2 C7198C2 C7214C2 C7239C2 C7247C2 C7256C2 C7280C2 C7292C2 C7300C2 C7353C2 C7356C2 C7380C2 C7397C2 C7398C2 C7432C2 C7450C2 C7465C2 C7496C2 C7499C2 C7501C2 C7518C2 C7521C2 C7551C2 C7573C2 C7621C2 C7623C2 C7650C2 C7651C2 C7656C2 C7657C2 C7672C2 C8024C2 C8067C2 C8103C2 C8104C2 C8201C2 C8202C2 C8211C2 C8252C2 C8277C2 C8306C2 C8351C2 C8369C2 C8395C2 C8397C2 C8399C2 C8400C2 C8412C2 C8413C2 C8431C2 C8440C2 C8441C2 C8480C2 C8495C2 C8502C2 C8511C2 C8517C2 C8541C2 C8559C2 C8567C2 C8569C2 C8574C2 C8576C2 C8596C2 C8605C2 C8609C2 C8623C2 C8634C2 C8649C2 C8667C2 C8670C2 C8732C2 C8754C2 C8756C2 C8757C2 C8769C2 C8770C2 C8784C2 C8794C2 C8797C2
Method	2	COA NIF

**Sample Dimensions Analysis**	
Covariance Parameters	3
Columns in X	3
Columns in Z	0
Subjects	110
Max Obs. per Subject	2

Convergence criteria met.

**Covariance Parameter Estimates**		
**Cov Parm**	**Subject**	**Estimate**
UN (1,1)	Lot	0.004338
UN (2,1)	Lot	0.002387
UN (2,2)	Lot	0.009730

**Fit Statistics**	
-2 Res Log Likelihood	−435.5
AIC (Smaller is Better)	−429.5
AICC (Smaller is Better)	−429.4
BIC (Smaller is Better)	−421.4

**Null Model Likelihood Ratio Test**		
**DF**	**Chi-Square**	**Pr > ChiSq**
2	29.98	<0.0001

**Solution for Fixed Effects**									
**Effect**	**Method**	**Estimate**	**Standard** **Error**	**DF**	***t* Value**	**Pr > |*t*|**	**Alpha**	**Lower**	**Upper**
Intercept		3.8555	0.01059	109	364.09	<0.0001	0.1	3.8379	3.8731
Method	COA	0.1297	0.01040	109	12.47	<0.0001	0.1	0.1124	0.1469
Method	NIF	0	.	.	.	.	.	.	.

**Type 3 Tests of Fixed Effects**				
**Effect**	**Num DF**	**Den DF**	**F Value**	**Pr > F**
Method	1	109	155.39	<0.0001

**Least Squares Means**									
**Effect**	**Method**	**Estimate**	**Standard** **Error**	**DF**	***t* Value**	**Pr > |*t*|**	**Alpha**	**Lower**	**Upper**
Method	COA	3.9852	0.006280	109	634.62	<0.0001	0.1	3.9747	3.9956
Method	NIF	3.8555	0.01059	109	364.09	<0.0001	0.1	3.8379	3.8731

**Differences of Least Squares Means**										
**Effect**	**Method**	**_Method**	**Estimate**	**Standard** **Error**	**DF**	***t* Value**	**Pr > |*t*|**	**Alpha**	**Lower**	**Upper**
Method	COA	NIF	0.1297	0.01040	109	12.47	<0.0001	0.1	0.1124	0.1469

The Mixed Procedure

Unit = 100

**Class Level Information**		
**Class**	**Levels**	**Values**
Lot	85	C4554C3 C4563C3 C4576C3 C4597C3 C4862C3 C4934C3 C4935C3 C5002C3 C5003C3 C5013C3 C5014C3 C5041C3 C5134C3 C5142C3 C5189C3 C5270C3 C5291C3 C5298C3 C5299C3 C5303C3 C5395C3 C5406C3 C5425C3 C5447C3 C5460C3 C5477C3 C5608C3 C5617C3 C5634C3 C5731C3 C5739C3 C5740C3 C5823C3 C5835C3 C5874C3 C6195C3 C6230C3 C6286C3 C6307C3 C6334C3 C6346C3 C6492C3 C6622C3 C6650C3 C6719C3 C6754C3 C6776C3 C6867C3 C6894C3 C6907C3 C6936C3 C7001C3 C7012C3 C7042C3 C7043C3 C7071C3 C7121C3 C7139C3 C7250C3 C7278C3 C7282C3 C7284C3 C7347C3 C7349C3 C7373C3 C7411C3 C7437C3 C7566C3 C7567C3 C7568C3 C7581C3 C7584C3 C7587C3 C7626C3 C7627C3 C7628C3 C7631C3 C7634C3 C7635C3 C7638C3 C7652C3 C7653C3 C8199C3 C8665C3 C8679C3
Method	2	COA NIF

**Dimensions**	
Covariance Parameters	3
Columns in X	3
Columns in Z	0
Subjects	85
Max Obs. per Subject	2

Convergence criteria met.

**Covariance Parameter Estimates**		
**Cov Parm**	**Subject**	**Estimate**
UN(1,1)	Lot	0.004874
UN(2,1)	Lot	0.001755
UN(2,2)	Lot	0.009360

**Fit Statistics**	
-2 Res Log Likelihood	−354.1
AIC (Smaller is Better)	−348.1
AICC (Smaller is Better)	−348.0
BIC (Smaller is Better)	−340.8

**Null Model Likelihood Ratio Test**		
**DF**	**Chi-Square**	**Pr > ChiSq**
2	14.53	0.0007

**Solution for Fixed Effects**									
**Effect**	**Method**	**Estimate**	**Standard** **Error**	**DF**	***t* Value**	**Pr > |*t*|**	**Alpha**	**Lower**	**Upper**
Intercept		4.5266	0.01067	84	424.19	<0.0001	0.1	4.5089	4.5444
Method	COA	0.1172	0.01140	84	10.28	<0.0001	0.1	0.09821	0.1361
Method	NIF	0	.	.	.	.	.	.	.

**Type 3 Tests of Fixed Effects**				
**Effect**	**Num DF**	**Den DF**	**F Value**	**Pr > F**
Method	1	84	105.66	<0.0001

**Least Squares Means**									
**Effect**	**Method**	**Estimate**	**Standard** **Error**	**DF**	**t Value**	**Pr > |*t*|**	**Alpha**	**Lower**	**Upper**
Method	COA	4.6438	0.007573	84	613.22	<0.0001	0.1	4.6312	4.6564
Method	NIF	4.5266	0.01067	84	424.19	<0.0001	0.1	4.5089	4.5444

**Differences of Least Squares Means**										
**Effect**	**Method**	**_Method**	**Estimate**	**Standard** **Error**	**DF**	***t* Value**	**Pr > |*t*|**	**Alpha**	**Lower**	**Upper**
Method	COA	NIF	0.1172	0.01140	84	10.28	<0.0001	0.1	0.09821	0.1361

**90% CI for Ratio of Geometric Means**

**Unit**	**DF**	**geomean_ratio**	**LowerCL**	**UpperCL**
50	109	113.843	111.896	115.825
100	84	112.431	110.320	114.583

**Geometric Mean and 95% CI for Each Method**

**Unit**	**Method**	**DF**	**geomean**	**LowerCL**	**UpperCL**
50	COA	109	3.98516	3.97474	3.99558
50	NIF	109	3.85551	3.83794	3.87308
100	COA	84	4.64380	4.63120	4.65639
100	NIF	84	4.52663	4.50888	4.54437

## Appendix B

**Table A1:** Cross-validation results of six batches.

Nominal Potency (U/vial)	Sample Number	Measured Potency (U/vial)	Accuracy by NIFDC CBPA (%)	Mean Measured Potency (U/vial0)	%CV	Measured Potency by NIFDC mLD50 (n = 1)	Mean Measured Potency by Allergan CBPA Release (n = 3)
50	C8399C2 (n = 5)	51	101	50	4.1	58	46
		52	103				
		48	95				
		52	104				
		48	96				
	C8441C2 (n = 5)	51	102	51	3.0	55	57
		51	101				
		52	104				
		48	96				
		51	102				
	C8596C2 (n = 5)	44	88	46	5.1	57	55
		48	96				
		45	89				
		46	91				
		50	99				
100	C7278C3 (n = 3)	90	90	106	14.0	93	95
		110	110				
		119	119				
	C8679C3 (n = 4)	97	97	97	1.0	112	103
		98	98				
		96	96				
		98	98				
	C8486C3 (n = 5)	102	102	104	1.7	111	107
		103	103				
		106	106				
		103	103				
		99	99				

**Table A2:** Preparation of reference standard and test sample.

Test Sample	Potency Level%	Nominal Value Unit/vial	Reconstitution Volume
WPRS04	100	103	400 μL reconstitution medium A
Test sample A	50	52	400 μL reconstitution medium A + 400 μL dilution medium
Test sample B	70	74	400 μL reconstitution medium A + 160 μL dilution medium
Test sample C	100	103	400 μL reconstitution medium A
Test sample D	130	129	320 μL reconstitution medium D
Test sample E	200	206	200 μL reconstitution medium E

Note: Test samples A, B, C, D, and E were prepared by WPRS04.

**Table A3:** Execution test plan of CBPA assay method validation.

Test Run	Analyst	
	A	B
1	3 96-well plates, 5 potency levels	3 96-well plates, 5 potency levels
2	3 96-well plates, 5 potency levels	3 96-well plates, 5 potency levels
3	3 96-well plates, 5 potency levels	3 96-well plates, 5 potency levels
4	6 96-well plates, 1 potency level (repeatability)	Repeat testing for the invalid tests from the previous two weeks if necessary
